# A multi‐centre prospective cohort study investigating the roles of psychological flexibility and self‐compassion in appearance concerns after burn injuries

**DOI:** 10.1111/bjhp.12754

**Published:** 2024-10-02

**Authors:** Laura Shepherd, Fuschia M. Sirois, Diana Harcourt, Paul Norman, David Aaron, Kate Adkins, Anna Cartwright, Emma Hodgkinson, Nicola Murphy, Andrew R. Thompson

**Affiliations:** ^1^ Nottingham University Hospitals NHS Trust Nottingham UK; ^2^ University of Sheffield Sheffield UK; ^3^ Durham University Durham UK; ^4^ University of the West of England Bristol UK; ^5^ The Mid Yorkshire Teaching NHS Trust Wakefield UK; ^6^ Sheffield Teaching Hospitals NHS Foundation Trust Sheffield UK; ^7^ Buckinghamshire Healthcare NHS Trust Aylesbury UK; ^8^ Department of Psychology, Institute of Psychiatry, Psychology and Neuroscience King's College London London UK; ^9^ The Newcastle upon Tyne Hospitals NHS Foundation Trust Newcastle UK; ^10^ Swansea Bay University Health Board Swansea UK; ^11^ Cardiff and Vale University Health Board and Cardiff University Cardiff UK

**Keywords:** appearance, body image, burn, injury, psychological flexibility, self‐compassion

## Abstract

**Objectives:**

Appearance concerns following burn injuries are common. Psychological factors are important in maintaining such concerns. However, there is a lack of longitudinal or prospective research investigating their development. This study investigated whether psychological flexibility and self‐compassion at hospital admission predicted subsequent appearance concerns.

**Design:**

A multi‐centre prospective cohort study across six burn services.

**Methods:**

Adults (*n* = 175; 67% male) in hospital following burn injuries were recruited. Questionnaires measuring appearance concerns, psychological flexibility, self‐compassion, post‐traumatic stress disorder symptoms and perceived noticeability were completed during hospital admission and two‐ and six‐months later. Demographic and burn injury information was collected.

**Results:**

Correlational analyses showed that increased psychological flexibility and self‐compassion at admission were associated with decreased appearance concerns cross‐sectionally and prospectively at two‐ and six‐month follow‐up. These associations remained significant when controlling for key covariates (i.e. gender, age, ethnicity, percentage total body surface area burnt, perceived noticeability, PTSD symptoms). Multiple linear regression analyses revealed that psychological flexibility and self‐compassion predicted appearance concerns during hospital admission. Although psychological flexibility significantly predicted appearance concerns over time, it became non‐significant when controlling for baseline appearance concerns.

**Conclusions:**

Psychological flexibility has a protective role against appearance concerns soon after burn injuries, although this protective role is reduced when accounting for baseline appearance concerns. Early interventions targeting psychological flexibility (i.e. acceptance and commitment therapy) may be beneficial after burns if adapted to address appearance‐related concerns.

**Statement of contribution**

**What is already known on this subject?**

- Psychological factors are important in maintaining appearance concerns in those with visible differences.
- Cross‐sectional research suggests that psychological flexibility and self‐compassion are related to appearance concerns.

**What does this study add?**

- Psychological flexibility and self‐compassion are related to appearance concerns following burn injuries, during hospital admission.
- Increased psychological flexibility during hospital admission after a burn injury predicts lower appearance concerns over time, although not over the effect of baseline appearance concerns.

## INTRODUCTION

Appearance concerns following burn injuries (Patterson et al., 1993) are common, as evidenced in people with other visible differences (Thompson & Kent, 2001). Thombs et al. (2008) were the first to track appearance concerns in burn patients over the first 12 months post‐injury. In this study, severity of appearance concerns was reported to be consistent over the 12 months but worse for female patients and those with larger burn injuries. Appearance concerns were also reported as the most salient predictor of psychosocial functioning after 12 months. Appearance concerns in the early post‐injury phase have been associated with long‐term disability and difficulty returning to work (Esselman et al., 2007). Therefore, understanding factors that have a role in the development and maintenance of, or protection from, appearance concerns is important.

### Psychological variables associated with appearance concerns after burns

Although some studies have reported a link between the severity of a burn injury (e.g. percentage total body surface area; %TBSA) and appearance concerns, a systematic review reported that findings vary significantly (Cleary et al., 2020), thereby indicating a potential role of psychological variables. However, there is only a small body of research that has examined the psychological variables associated with appearance concerns following burn injuries. Cross‐sectional studies have highlighted the role of shame in social introversion due to scarring (Taal & Faber, 1998) and social stigma, post‐traumatic stress and depression symptoms as mediators of the relationship between appearance concerns and reduced community integration (Mercado et al., 2022). Considering longitudinal or prospective cohort studies, three studies from the same research group in North America have highlighted relationships between increased use of emotion‐focused coping (Fauerbach et al., 2002) and avoidant coping (Amoyal et al., 2011), and higher appearance salience (Thombs et al., 2008), during hospital admission and subsequent appearance concerns between two and 12 months later.

Coping strategies can be seen as being driven by broader constructs relating to how individuals respond to internal experiences (e.g. emotions and thoughts), such as psychological flexibility (Hayes et al., 1999) and self‐compassion (Neff, 2003). These broader constructs may themselves be related to appearance concerns after burns and be suitable targets for intervention. Indeed, psychological flexibility (Hayes et al., 1999) and self‐compassion (Neff, 2003) have been found to be related to reduced shame and guilt, as well as higher levels of adaptive coping, in various clinical and medical populations (Sirois et al., 2015; Vasiliou et al., 2023; Williams et al., 2021; Zukerman et al., 2023). Psychological flexibility and self‐compassion may therefore also help to buffer the impact of burn injuries on appearance concerns.

### Psychological flexibility and appearance concerns

Psychological flexibility is a multi‐dimensional theory of psychological wellbeing underpinning acceptance and commitment therapy (ACT; Hayes et al., 1999) and can be defined as being open to experiencing distressing internal experiences (e.g. thoughts and emotions), being present and acting in line with an individual's values in spite of their distressing experiences (Harris, 2009). In contrast, experiential avoidance (difficulties being open to experiencing distressing internal experiences) is associated with psychological difficulties (Hayes et al., 1999). A diathesis‐stress model of experiential avoidance has been proposed, suggesting that experiential avoidance makes people vulnerable to stressors, in that individuals who typically try to avoid internal distressing experiences may continue this approach when faced with stressful life events (Biglan et al., 2008).

There has been recent interest in the roles of psychological flexibility (or facets of this construct) in appearance concerns in the wider visible difference literature (Montgomery et al., 2016; Shepherd et al., 2019; Zucchelli, White, & Williamson, 2020). Mindfulness, which relates to the being present element of psychological flexibility, was found to explain 41% of the variance in social anxiety in a sample of 120 individuals with skin conditions (Montgomery et al., 2016). Furthermore, in a sample of 78 individuals who had experienced burn injuries, numerous facets of psychological flexibility (acceptance, cognitive defusion, mindfulness and valued action) were found to be associated with appearance concerns (Shepherd et al., 2019). In a further study by Zucchelli, White, and Williamson (2020), with 220 adults with varying visible differences, cognitive fusion (difficulty standing back from and observing thoughts) was found to partially mediate the relationship between body evaluation and body image coping strategies. In addition, acceptance was reported to partially mediate the relationship between body evaluation and behavioural avoidance. Finally, Vasiliou et al. (2023) explored stigma in 105 individuals with visible skin conditions, finding that psychological flexibility contributed to fewer stigmatized experiences.

### Self‐compassion and appearance concerns

Neff (2003) defines self‐compassion as the ability to respond to difficulties and personal struggles with self‐kindness rather than harsh self‐criticism, see one's experience as part of a common humanity rather than feeling alone in one's suffering and take a balanced perspective on painful thoughts and emotions rather than becoming immersed in them. Together, these three bipolar components of self‐compassion work synergistically to defuse negative thoughts and responses to difficulties, promote self‐acceptance and help feel less isolated, which in turn can reduce stress, promote adaptive coping and improve well‐being (Ewert et al., 2021; Sirois et al., 2015; Zessin et al., 2015). The limited research on the possible role of self‐compassion in appearance concerns relating to visible differences has mainly been conducted with breast cancer survivors. One study reported that self‐compassion mediated the relationship between negative body image and distress (Przezdziecki et al., 2013). In other studies, higher self‐compassion has been linked to lower body image difficulties (Todorov et al., 2019; Zhu et al., 2023). A further study found that the association between having a negative body image and psychological distress in women following nipple‐sparing mastectomy and immediate breast reconstruction was attenuated by higher levels of self‐compassion (Sherman et al., 2017).

There is also some research with individuals with skin conditions that supports a potential protective role for self‐compassion in appearance concerns. Firstly, although not measuring appearance concerns or body image disturbance specifically, one study exploring the effect of self‐compassion on symptoms of depression in patients with skin conditions found that self‐compassion explained significant variance in depression and moderated the effect on disgust propensity (Clarke et al., 2020). Secondly, a qualitative study by the same research group reported that self‐compassion helped people adjust to living with a skin condition (Clarke et al., 2022).

### Study rationale and aims

Current theory and evidence suggests that psychological flexibility and self‐compassion may be protective factors in appearance concerns in individuals with visible differences. However, research is predominantly limited to cross‐sectional studies, which limits conclusions about the roles of psychological flexibility and self‐compassion in the maintenance rather than the development of appearance concerns. Longitudinal or prospective studies are therefore required to assess appearance concerns and psychological variables in the early stages following a burn injury (e.g. during hospital admission) and repeat these over time. Such studies can help identify the psychological variables that increase risk for, and protection from, appearance concerns.

Furthermore, evidence related to appearance concerns is currently limited to studies that have investigated psychological flexibility and self‐compassion separately. There is evidence that these constructs are empirically related (Moreno, 2023; Neff & Tirch, 2013). Moreover, Yadavaia et al. (2014) have argued that psychological flexibility and self‐compassion are also conceptually related. First, Yadavaia et al. (2014) argued that Neff's (2003) concept of self‐kindness may be closely associated with self‐acceptance, the opposite of experiential avoidance. This is because self‐acceptance, through acceptance of internal experiences and reducing experiential avoidance, is likely to facilitate self‐compassion. Second, they argued that the same processes that enhance perspective taking (or self‐as‐context; an awareness of the self as separate to one's internal experiences) in psychological flexibility are involved in a sense of common humanity (central to Neff's conceptualization of self‐compassion), as they allow individuals to appreciate that both the self and others have moment‐to‐moment perspectives that involve being witnesses to difficult experiences. Third, they argued that both concepts emphasize mindfulness by defusion, acceptance, contact with the present moment and self‐as‐context in psychological flexibility and the ability to have a balanced perspective on painful thoughts and emotions rather than be overwhelmed by them in Neff's conceptualization of self‐compassion.

Therefore, investigating both psychological flexibility and self‐compassion together would generate insight into whether each plays a unique role in appearance concerns or whether one plays a stronger role than the other. It would also provide clear clinical implications as to the type of psychological intervention that is likely to be most effective in reducing or preventing appearance concerns following burn injuries.

The present study therefore aimed to investigate whether psychological flexibility and self‐compassion during hospital admission were associated with appearance concerns both cross‐sectionally and prospectively at two‐ and six‐month follow‐up in adults with burn injuries. We hypothesized that higher levels of psychological flexibility and self‐compassion during hospital admission would predict lower appearance concerns at baseline as well as at the follow‐ups.

## MATERIALS AND METHODS

### Design

A multi‐centre prospective cohort study was conducted. Standardized self‐report questionnaires were administered across three time points: during hospital admission (T1), 2 months later (T2) and 6 months later (T3). The independent variables were psychological flexibility and self‐compassion. The dependent variable was appearance concerns. Seven covariates were identified a priori based on previous studies that have found them to be associated with appearance concerns (Cleary et al., 2020; Macleod et al., 2016; Mata‐Greve et al., 2022; Montgomery et al., 2016; Shepherd et al., 2019; Thombs et al., 2008), namely, age, gender, ethnicity, burn severity (measured by percentage total body surface area burnt (% TBSA)), post‐traumatic stress disorder (PTSD) symptoms, perceived noticeability and baseline appearance concerns.

### Power analysis

An a priori power analysis using the fixed model, *R* ^2^ deviation from zero test provided by G*Power, indicated that data from at least 114 participants would be required to detect a statistically significant result at *p* = .05 based on 80% power and an effect size of *f* ^2^ = .15 with nine predictor variables. A medium effect size of *f* ^2^ = .15, according to Cohen's (1992) guidelines, was chosen given the lack of previous research on psychological flexibility and self‐compassion in relation to appearance concerns following burn injuries. A systematic review and meta‐analysis estimated the mean retention rate in longitudinal cohort studies to be 73.5%, although females had a higher (78.5%) retention rate than males (60.1%; Teague et al., 2018). Given that more adult males (63.6%) than females (36.4%) are admitted with burn injuries (Mehta et al., 2022), the expected retention rate was estimated to be 67%. This suggested recruiting 170 participants at baseline. To account for incomplete data, 175 participants were recruited.

### Participants

Adults (*n* = 175) admitted to the hospital with a burn injury were recruited. Males represented 66.9% (*n* = 117) of the sample, and 33.1% (*n* = 58) were female. The mean age was 42.2 years (range: 18–86; *SD* = 16.35). The majority were White British (82.9%; *n* = 145) and 17.1% (*n* = 30) were from other ethnic groups. The mean self‐reported socio‐economic status, measured by the *McArthur Scale of Subjective Social Status – Adult Version* (Adler et al., 2000), was 5.90 (*SD* = 1.58), where one represents the ‘worst off’ people and 10 represents the ‘best off’ people in the UK. Table 1 displays burn injury information.

**TABLE 1:** Participant burn injury information.

	Mean (SD)
Days since burn injury at recruitment	8.7 (15.21)
% Total body surface area (TBSA)	5.8 (7.68)
	***N* (%)**
Mechanism	
Scald	61 (34.9%)
Flame	46 (26.3%)
Flash	25 (14.3%)
Contact	20 (11.4%)
Chemical	13 (7.4%)
Electrical	4 (2.3%)
Radiation	2 (1.1%)
Other	4 (2.3%)
Depth	
Superficial partial thickness	64 (36.6%)
Mixed thickness	59 (33.7%)
Full thickness	40 (22.9%)
Deep dermal	12 (6.9%)
Body location	
Legs	87 (49.7%)
Arms	60 (34.3%)
Hands	46 (26.3%)
Chest/abdomen	45 (25.7%)
Feet	38 (21.7%)
Head/face/neck	31 (17.7%)
Back	16 (9.1%)
Buttocks	10 (5.7%)
Genitals	8 (4.6%)

As Table 1 indicates, participants were recruited on average less than 9 days following their burn, and the mean TBSA was 5.8%. The most common areas burnt on the body were the legs (49.7%), but the sample included those with burn injuries across a variety of body areas.

### Inclusion and exclusion criteria

Adults (18 years of age or older) who were inpatients at recruiting burn services with acute burn injuries were eligible to participate. Individuals were excluded if they had been admitted for reconstructive surgery rather than an acute injury, were too physically unwell, sustained their burn injuries due to a suicide attempt or self‐harm, were not fluent in the English language, had a cognitive impairment, or had no potential ability to participate by completing questionnaires at all time points (i.e. no contact details).

### Settings

Six designated specialized NHS burns units or centres in Aylesbury, Newcastle, Nottingham, Sheffield and Wakefield, England, and Swansea, Wales, UK, recruited participants. The number of participants recruited between the sites varied (range: 8–87). Considering representativeness of the sample, the proportion of participants taking part in the study compared to the total number of burn patients admitted to each burn service due to accidental burn injuries during the recruitment period ranged from 7% to 25.4% across the recruitment sites, with an average of 12.3%.

### Measures

During hospital admission (T1), participants completed four questionnaires and two single‐item visual analogue scales.

#### Body Esteem for Adolescents and Adults – Appearance Subscale (BESAA‐A)

Body Esteem for Adolescents and Adults – Appearance Subscale (BESAA‐A; Mendelson et al., 2001): the 10‐item appearance subscale of this self‐report questionnaire that measures body esteem and evaluation was used to measure appearance concerns. It has been used in research exploring psychological variables and appearance concerns in adults following burns, with an alpha coefficient of .95 reported (Lawrence et al., 2006), and to assess the relationship between body esteem and psychological flexibility in participants with a range of visible differences (Zucchelli, White, & Williamson, 2020). Responses to items are rated on a scale ranging from 0 (‘Not at all’) to 4 (‘Always’), with some items being reverse‐scored. An example item is, ‘I'm pretty happy about the way I look’. The total mean score of this measure was used in analyses, which has a possible range of values between 0 and 4. Higher scores indicate higher body‐esteem (lower appearance concerns).

#### Acceptance and Action Questionnaire (AAQ‐II)

Acceptance and Action Questionnaire (AAQ‐II; Bond et al., 2011): a seven‐item self‐report questionnaire that measures difficulties with acceptance, one facet of psychological flexibility. It has good psychometric properties, with an alpha coefficient of .84 and 3‐ and 12‐month test–retest reliability of .81 and .79, respectively. It is the most widely used measure in psychological flexibility research (Ong et al., 2020) and has been used in a cross‐sectional study exploring psychological flexibility and appearance concerns in adults following burn injuries (Shepherd et al., 2019). Responses to items are rated on a scale ranging from 1 (‘Never true’) to 7 (‘Always true’). An example item is, ‘I'm afraid of my feelings’. The total score of this measure was used in analyses, which has a possible range of values between 7 and 49. Higher scores indicate higher experiential avoidance, and therefore lower psychological flexibility.

#### Self‐Compassion Scale – Short Form (SCS‐SF)

Self‐Compassion Scale – Short Form (SCS‐SF; Raes et al., 2011): a 12‐item self‐report questionnaire that measures self‐compassion. It has good psychometric properties, with an alpha coefficient of .86 (Raes et al., 2011), and has been widely used in research examining self‐compassion and body image in populations with a visible difference (Sherman et al., 2017). Responses to items are rated on a scale ranging from 1 (‘Almost never’) to 5 (‘Almost always’). An example item is, ‘I try to be understanding and patient towards those aspects of my personality I don't like’. The total mean score of this measure was used in analyses, which has a possible range of values between 1 and 5. Higher scores indicate higher self‐compassion.

#### Impact of Event Scale – Revised (IES‐R)

Impact of Event Scale – Revised (IES‐R; Weiss & Marmar, 1997): a 22‐item self‐report questionnaire that measures post‐traumatic stress disorder (PTSD) symptoms associated with a traumatic event. It has good psychometric properties, with an alpha coefficient of .96, and has been widely used in trauma research, including burn injury populations (Shepherd et al., 2019). Responses to items are rated on a scale ranging from 0 (‘Not at all’) to 4 (‘Extremely’). An example item is, ‘Pictures about it popped into my mind’. The total score of this measure was used in analyses, which has a possible range of values between 0 and 88. Higher scores indicate increased PTSD symptoms.

#### Perceived noticeability

This has been found to be associated with appearance concerns in previous research (Montgomery et al., 2016). Perceived noticeability has been measured and controlled for in previous research that studied a sample of adults with a range of visible differences using visual analogue scales (Zucchelli, White, & Williamson, 2020). The same scale was used in this study with slightly amended instructions to measure participants' perceptions about how noticeable the appearance of their burn injuries will be to other people after discharge from hospital. Participants rate this on a scale of 0 to 10, where 0 represents ‘Not at all noticeable’ and 10 represents ‘Very noticeable’.

#### McArthur Scale of Subjective Social Status – Adult Version

McArthur Scale of Subjective Social Status – Adult Version (Adler et al., 2000): Participants also completed this single‐item scale, a validated and well‐known measure of perceived socio‐economic status. Participants rank themselves on a ladder labelled from one (‘Worst off’) to 10 (‘Best off’), representing their socio‐economic standing compared with other people in the UK. These data were used to contextualize the sample.

#### Demographic and burn injury information

Additional demographic information (gender, age and ethnicity) and burn injury details (mechanism of burn injury, percentage of total body surface area burnt (%TBSA), depth of burn, location(s) of burns on the body and number of days from burn injury to participation) were obtained from participants' medical records. Data were used to contextualize the sample.

### Procedure

Recruitment took place between January 2021 and September 2022. Potential participants were approached during their hospital admission if they were deemed to meet inclusion criteria by a clinical psychologist or assistant psychologist working in the service. Participants were assigned a unique four‐digit identifier to link data across the time points. Participants chose how they would prefer to be contacted at the subsequent data collection time points (by post, email with a Qualtrics link, telephone/video call). Participants completed the questionnaires and visual analogue scales prior to discharge from the hospital. Participants were then contacted 2 months (T2) and 6 months (T3) later, using the participant's preferred contact method(s), and prompted to complete the questionnaires and rating scales. Participants were offered a £10 voucher for participation at all time points.

Two months and six months later (T2 and T3), participants completed the Body Esteem for Adolescents and Adults – Appearance Subscale (BESAA‐A), Acceptance and Action Questionnaire (AAQ‐II), Self‐Compassion Scale – Short Form (SCS‐SF) and Impact of Event Scale – Revised (IES‐R) questionnaires again, in addition to the visual analogue scale measuring perceived noticeability. At these time points, the perceived noticeability visual analogue scale instruction was amended to ask participants to consider how noticeable they believed their burn injuries were to other people at that time, in line with the instructions given by Zucchelli, White, and Williamson (2020).

### Sampling

Purposive sampling was used, aided by an inclusion/exclusion criteria checklist. The sample within this study represented, on average, 12.3% of all burn patients being admitted to the participating burn services due to accidental burn injuries during the recruitment period.

### Ethical approval

The study obtained ethical approval from the London – Dulwich NHS Research Ethics Committee (REC) and Health Research Authority (IRAS ID: 282011; REC ref. 20/PR/0838) in January 2021. The sponsor of the study was Nottingham University Hospitals NHS Trust (ref. 20CP003). Research and development (R&D) approval was gained from all NHS trust recruitment sites prior to recruitment starting. The study was also registered by the University of Sheffield (ref. 171289).

### Data analysis

The Statistical Package for the Social Sciences (SPSS) version 26 was used to analyse the data. Alpha (the risk of making a type I error) was set at .05. Accordingly, *p* values ≤ .05 were taken to be statistically significant. Independent *t*‐tests and Pearson's chi square analyses were used to explore differences or associations between participants who were retained throughout the study versus lost to follow up at the two‐ and six‐month data collection points (i.e. attrition). Little's ‘missing completely at random’ (MCAR) tests suggested that missing data were MCAR at T1 (*χ* ^2^ = 330.65, *df* = 968, *p* = 1.00), T2 (*χ* ^2^ = 209.11, *df* = 469, *p* = 1.00) and T3 (*χ* ^2^ = 308.45, *df* = 448, *p* = 1.00) based on all questionnaire and visual analogue scale data. The amount of missing data was small (T1: .6%–4%; T2: 0%–1.6%; T3: 0%–2.5%). Expectation maximization was used for missing questionnaire values for participants not lost to follow‐up. If any questionnaire had 20% or more missing items, the data for that scale were not used. All analyses were completed with missing data values inputted.

Descriptive statistics and repeated measures ANOVAs were conducted to compare the scores on the measures across the time points to analyse whether scores had changed over time. Pearson's correlation analyses were conducted to analyse the relationships between both psychological flexibility and self‐compassion and appearance concerns at each time point. Pearson's correlation analyses were also used to assess relationships between participants' demographic and burn details and appearance concerns at each time point. Partial correlational analyses were then used to assess associations between psychological flexibility, self‐compassion and appearance concerns while controlling for covariates (age, gender, ethnicity, % TBSA, PTSD symptoms and perceived noticeability). The covariates of PTSD symptoms and perceived noticeability were taken from the same time point for the cross‐sectional analyses and from T1 for the prospective analyses. Multiple linear regression analyses were conducted to explore how much of the variance in appearance concerns at T1, T2 and T3 was explained by the covariates (block 1), T1 psychological flexibility and self‐compassion (block 2) and where prospective analyses were undertaken to predict T2 and T3 appearance concerns, T1 appearance concerns (block 3). There are no assumptions regarding the (normal) distribution of the independent variables for multiple linear regression (Tabachnick & Fidell, 2019). Instead, the distribution of the residuals (i.e., linearity, normality and homoscedasticity) should be examined. Histograms, PP plots and scatterplots of residuals of the above three regression analyses were therefore inspected. These indicated that the statistical assumptions for the regression analyses had been met.

Inspection of histograms and QQ plots suggested that a number of variables were not normally distributed: age; % TBSA; AAQ‐II scores; IES‐R scores; and perceived noticeability scores. Box plots suggested that perceived noticeability scores at T2 and T3 and % TBSA had outliers that could have impacted the data. Given that multiple linear regression analyses were the main analyses in the study and these do not rely on these assumptions, parametric analyses were used throughout for consistency.

The data that support the findings of this study are openly available in the Open Science Framework at https://doi.org/10.17605/OSF.IO/T8SJ4. Impairment in work and social functioning, as well as Covid‐19‐related distress, were also measured but are not reported here. Information and analyses related to these variables can be found in supplementary materials that are hosted on the Open Science Framework.

## RESULTS

### Participant recruitment and attrition

**FIGURE 1:**
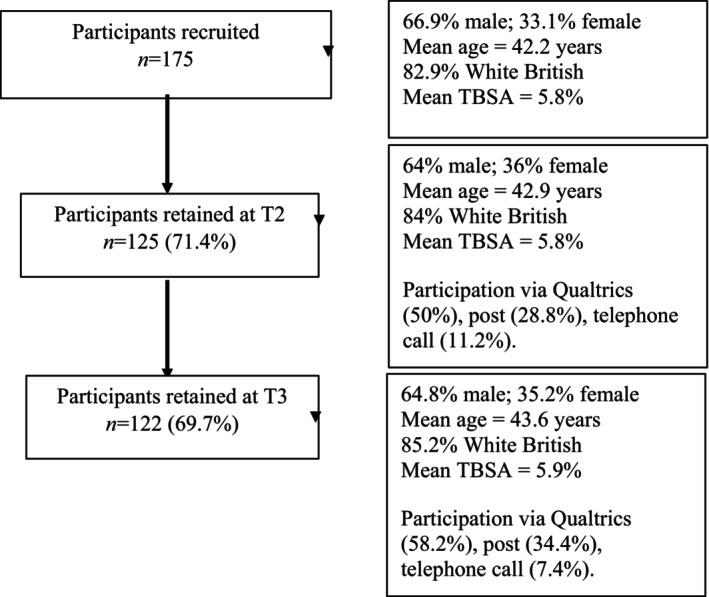
Participants recruited and retained.

Figure 1 displays the number of participants recruited at T1 and retained at T2 and T3. Key demographic, burn injury and method of participation information are presented.

There were no significant differences between participants retained versus those lost at two‐ or six‐month follow‐up on measures of demographics (gender, age, ethnicity, socioeconomic status), clinical characteristics (%TBSA, recruitment site) or scores on measures of appearance concerns (BESAA‐A), psychological flexibility (AAQ‐II), self‐compassion (SCS‐SF), PTSD symptoms (IES‐R) and perceived noticeability (visual analogue scale).

### Changes in appearance concerns, psychological flexibility and self‐compassion and covariates over time

The means and standard deviations of measures used in analyses at each time point are displayed in Table 2 along with change statistics. There was a significant effect of time on appearance concerns. However, there were no statistically significant differences between appearance concerns at T1 compared to T2 (mean difference = .20, *p* = .06), between T1 and T3 (mean difference = .15, *p* = .18) or between T2 and T3 (mean difference = −.05, *p* = 1.000). Post‐hoc power analyses revealed these comparisons were underpowered (power ranged from 17% to 68%). Psychological flexibility did not change over time. However, self‐compassion did change over time. Participants had higher self‐compassion scores at T1 compared to both T2 (mean difference = .30, *p* < .001) and T3 (mean difference = .26, *p* < .001), but not between T2 and T3 (mean difference = −.03, *p* = 1.00). PTSD symptoms were lower at T1 compared to T2 (mean difference = −4.50, *p* = .05), and higher at T2 compared to T3 (mean difference = 3.71, *p* = .003).

**TABLE 2:** Means (M), standard deviation (SD) of measures at each time point.

	M ± SD (range)			Change statistic
	T1	T2	T3	
Appearance concerns	2.58 ± .97 (.1–4.0)	2.38 ± 1.02 (.2–4.0)	2.43 ± 1.03 (.1–4.0)	*F*(1.57, 169.06) = 4.08, *p* = .03*
Psychological flexibility	19.35 ± 11.08 (7–49)	21.19 ± 11.69 (7–45)	20.20 ± 10.55 (7–43)	*F*(1.85, 201.17 = 2.20, *p* = .12)
Self‐compassion	3.30 ± .86 (1.5–4.83)	3.01 ± .87 (1.3–5)	3.04 ± .88 (1.1–5)	*F*(1.71, 186.04) = 14.29, *p* < .001***
PTSD symptoms	25.35 ± 20.32 (0–82)	30.24 ± 21.32 (0–76)	26.15 ± 20.04 (0–77)	*F*(1.58, 170.13) = 4.61, *p* = .02*
Perceived noticeability	2.44 ± 3.08 (0–10)	2.58 ± 3.17 (0–10)	2.06 ± 2.89 (0–10)	*F*(1.82, 194.23) = 1.49, *p* = .23

* *p* < .05.

*** *p* < .001.

### Relationships with appearance concerns

As shown in Table 3, lower appearance concerns were associated with increased psychological flexibility and self‐compassion cross‐sectionally at all time points. These correlations are large, according to Cohen (1992). Negative correlations between appearance concerns and psychological flexibility reflect the relationship between increased body esteem scores on the BESAA‐A (Mendelson et al., 2001) and lower experiential avoidance scores on the AAQ‐II (Bond et al., 2011). Positive correlations between appearance concerns and self‐compassion reflect the relationship between increased body esteem scores on the BESAA‐A (Mendelson et al., 2001) and increased self‐compassion scores on the SCS‐SF (Raes et al., 2011). Lower appearance concerns were also associated with lower PTSD symptoms (with medium or large effect sizes), lower perceived noticeability (with medium effect sizes) and male gender (with small or medium effect sizes) at all time points. In addition, older age was associated with decreased appearance concerns at T2 and T3, but not T1 (with small effect sizes). Ethnicity and % TBSA were not associated with appearance concerns at any time point.

**TABLE 3:** Relationships with appearance concerns at each time point.

	T1 appearance concerns	T2 appearance concerns	T3 appearance concerns
Gender	−.26***	−.27***	−.31***
Age	.15	.20*	.20*
Ethnicity	−.01	.04	.02
%TBSA	−.07	−.07	−.17
T1 Psychological flexibility	−.54***	−.51***	−.57***
T1 Self‐compassion	.50***	.45***	.51***
T1 PTSD symptoms	−.47***	−.41***	−.47***
T1 Perceived noticeability	−.37***	−.20*	−.21*
T2 Psychological flexibility	−.52***	−.73***	−.71***
T2 Self‐compassion	.53***	.68***	.69***
T2 PTSD symptoms	−.39***	−.59***	−.55***
T2 Perceived noticeability	−.16	−.32***	−.24**
T3 Psychological flexibility	−.54***	−.72***	−.79***
T3 Self‐compassion	.52***	.66***	.70***
T3 PTSD symptoms	−.41***	−.63***	−.61***
T3 Perceived noticeability	−.18	−.38***	−.35***

Abbreviation: %TBSA, percentage total body surface area burnt.

* *p* < .05.

** *p* < .01.

*** *p* < .001.

Prospectively, increased psychological flexibility and self‐compassion and decreased PTSD symptoms and perceived noticeability at T1 were related to lower appearance concerns at T2 and T3. The correlations between psychological flexibility and appearance concerns represent large effect sizes. The correlations between self‐compassion and appearance concerns represent a medium effect size at T2 and a large effect size at T3 (Cohen, 1992). The correlations between PTSD symptoms and appearance concerns represent medium effect sizes. The correlations between perceived noticeability and appearance concerns represent small effect sizes (Cohen, 1992). In addition, large‐sized correlations were found between psychological flexibility and self‐compassion at T1, *r*(172) = −.78, *p* < .001, T2, *r*(123) = −.72, *p* < .001 and T3, *r*(121) = −.70, *p* < .001.

As shown in Table 4, associations between the dependent and independent variables remained significant when controlling for covariates (gender; age; ethnicity; % TBSA; PTSD symptoms; perceived noticeability). Effect sizes were medium or large, with the exception of a small effect size related to the association between T1 self‐compassion and T2 appearance concerns (Cohen, 1992).

**TABLE 4:** Partial correlations between appearance concerns and psychological flexibility and self‐compassion, controlling for covariates.

	T1 appearance concerns	T2 appearance concerns	T3 appearance concerns
T1 Psychological flexibility	−.41***	−.35***	−.41***
T1 Self‐compassion	.40***	.26**	.36***
T2 Psychological flexibility		−.55***	−.51***
T2 Self‐compassion		.55***	.58***
T3 Psychological flexibility			−.62***
T3 Self‐compassion			.62***

** *p* < .01.

*** *p* < .001.

### Predicting appearance concerns

The predictive value of T1 psychological flexibility and self‐compassion on appearance concerns at T1, T2 and T3 was investigated. Table 5 presents the predictors of appearance concerns and multiple linear regression model statistics across block 1 (covariates) and block 2 (inclusion of psychological flexibility and self‐compassion) at T1.

**TABLE 5:** Hierarchical regression analysis predicting appearance concerns at T1.

Variable	*B*	SE	95% CI		*p*
			LL	UL	
Block 1					
Gender	−.386	.126	−.635	−.138	.03
Perceived noticeability	−.081	.020	−.119	−.042	<.001
PTSD symptoms	−.016	.003	−.022	−.011	<.001
Age	−.001	.004	−.008	.007	.86
Ethnicity	.154	.204	−.249	.557	.45
% TBSA	.002	.008	−.013	.016	.84
Block 2					
Gender	−.330	.114	−.555	−.104	.01
Perceived noticeability	−.099	.018	−.134	−.063	<.001
PTSD symptoms	−.004	.003	−.011	.002	.202
Age	−.002	.004	−.009	.005	.560
Ethnicity	.017	.186	−.351	.386	.926
% TBSA	.003	.007	−.011	.016	.682
Psychological flexibility	−.022	.008	−.038	−.006	.01
Self‐compassion	.229	.102	.027	.431	.03

Abbreviations: CI, confidence interval; LL, lower limit; SE, standard error; UL, upper limit.

At T1, the analysis revealed that the covariates explained 33% of the variance (*R* ^2^ = .33). The model was statistically significant, *F*(6, 158) = 12.99, *p* < .001. Male gender, decreased perceived noticeability and PTSD symptoms significantly predicted lower appearance concerns. Adding in T1 psychological flexibility and self‐compassion explained a further 13.2% of the variance in T2 appearance concerns (Δ*R* ^2^ = .13, Δ*F* = 19.08, *p* < .001). This model was also statistically significant, *R* ^2^ = .46, *F*(8, 156) = 16.74, *p* < .001. Male gender, decreased perceived noticeability and increased psychological flexibility and self‐compassion significantly predicted lower appearance concerns.

Tables 6 and 7 present the predictors of appearance concerns and multiple linear regression model statistics across block 1 (covariates), block 2 (inclusion of psychological flexibility and self‐compassion) and block 3 (baseline appearance concerns) at T2 and T3, respectively.

**TABLE 6:** Hierarchical regression analysis predicting appearance concerns at T2.

Variable	*B*	SE	95% CI		*p*
			LL	UL	
Block 1					
Gender	−.442	.177	−.792	−.091	.01
Perceived noticeability	−.023	.028	−.079	.033	.423
PTSD symptoms	−.017	.004	−.025	−.008	<.001
Age	.006	.006	−.005	.017	.275
Ethnicity	.392	.313	−.228	1.012	.213
% TBSA	7.662E‐5	.010	−.020	.020	.994
Block 2					
Gender	−.387	.167	−.718	−.056	.02
Perceived noticeability	−.034	.027	−.088	.020	.215
PTSD symptoms	−.005	.005	−.015	.005	.325
Age	.005	.005	−.006	.015	.396
Ethnicity	.261	.301	−.335	.857	.387
% TBSA	.000	.010	−.019	.019	.992
Psychological flexibility	−.038	.014	−.065	−.010	.01
Self‐compassion	−.031	.175	−.378	.316	.861
Block 3					
Gender	−.244	.157	−.005	.015	.317
Perceived noticeability	.016	.027	−.038	.070	.559
PTSD symptoms	−.002	.005	−.012	.007	.614
Age	.005	.005	−.005	.015	.317
Ethnicity	.234	.276	−.314	.782	.399
% TBSA	−.002	.009	−.019	.016	.839
Psychological flexibility	−.016	.013	−.043	.010	.228
Self‐compassion	.002	.161	−.317	.322	.990
T1 Appearance concerns	.492	.108	.279	.705	<.001

Abbreviations: CI, confidence interval; LL, lower limit; SE, standard error; UL, upper limit.

**TABLE 7:** Hierarchical regression analysis predicting appearance concerns at T3.

Variable	*B*	SE	95% CI		*p*
			LL	UL	
Block 1					
Gender	−.555	.178	−.908	−.202	.002
Perceived noticeability	−.017	.028	−.073	.038	.537
PTSD symptoms	−.019	.004	−.028	−.010	<.001
Age	.002	.006	−.009	.013	.689
Ethnicity	.421	.317	−.207	1.050	.187
% TBSA	−.013	.010	−.033	.007	.206
Block 2					
Gender	−.526	.165	−.853	−.199	.002
Perceived noticeability	−.029	.026	−.081	.023	.269
PTSD symptoms	−.006	.005	−.016	.003	.181
Age	.000	.005	−.011	.010	.938
Ethnicity	.311	.291	−.266	.888	.287
% TBSA	−.010	.009	−.028	.008	.278
Psychological flexibility	−.030	.013	−.056	−.004	.03
Self‐compassion	.162	.169	−.174	.497	.341
Block 3					
Gender	−.375	.150	−.674	−.077	.01
Perceived noticeability	.032	.026	−.019	.084	.219
PTSD symptoms	−.004	.004	−.013	.004	.347
Age	−.2.303E‐5	.005	−.009	.009	.996
Ethnicity	.274	.261	−.243	.791	.295
% TBSA	−.011	.008	−.027	.005	.181
Psychological flexibility	−.012	.012	−.037	.012	.324
Self‐compassion	.103	.152	−.198	.404	.499
T1 Appearance concerns	.521	.100	.323	.718	<.001

Abbreviations: CI, confidence interval; LL, lower limit; SE, standard error; UL, upper limit.

At T2, the covariates explained 22.5% of the variance in T2 appearance concerns (*R* ^2^ = .23). The model was statistically significant, *F*(6, 111) = 5.38, *p* < .001. Male gender and decreased PTSD symptoms significantly predicted lower appearance concerns. Adding in T1 psychological flexibility and self‐compassion explained a further 10% of the variance (Δ*R* ^2^ = .10, Δ*F* = 8.08, *p* < .001), and this model was statistically significant, *F*(8, 109) = 6.57, *p* < .001. In this model, male gender and increased psychological flexibility significantly predicted lower appearance concerns, whereas the *B* value for self‐compassion was non‐significant. Adding in T1 appearance concerns explained a further 10.9% of the variance (Δ*R* ^2^ = .11, Δ*F* = 20.89, *p* < .001), and this model was statistically significant, *F*(9, 108) = 9.22, *p* < .001. However, only lower T1 appearance concerns significantly predicted lower T2 appearance concerns.

At T3, the covariates explained 29.3% of the variance in T3 appearance concerns (*R* ^2^ = .29). The model was statistically significant, *F*(6, 108) = 7.44, *p* < .001. Male gender and decreased PTSD symptoms significantly predicted lower appearance concerns. Adding in T1 psychological flexibility and self‐compassion explained a further 12.7% of the variance (Δ*R* ^2^ = .13, Δ*F* = 11.58, *p* < .001), and this model was statistically significant, *F*(8, 106) = 9.57, *p* < .001. In this model, male gender and increased psychological flexibility significantly predicted lower appearance concerns, whereas the *B* value for self‐compassion was non‐significant. Adding in T1 appearance concerns explained a further 12% of the variance (Δ*R* ^2^ = .12, Δ*F* = 27.34, *p* < .001), and this model was statistically significant, *F*(9, 105) = 13.66, *p* < .001. In this model, only male gender and lower T1 appearance concerns significantly predicted lower T3 appearance concerns.

## DISCUSSION

This study prospectively investigated psychological flexibility and self‐compassion as protective factors against appearance concerns after burn injuries. There are four key findings.

First, increased psychological flexibility and self‐compassion were related to lower appearance concerns. This relationship was observed at all time points (hospital admission, 2 months later and 6 months later) and was sustained when controlling for demographic and clinical variables. Prospectively, correlation analyses indicated that higher levels of psychological flexibility and self‐compassion measured during hospital admission were associated with lower appearance concerns at the two and six month time points, and these relationships remained when controlling for demographic and clinical variables. However, it should be noted that the multiple regression analyses revealed that when controlling for psychological flexibility (as well as baseline appearance concerns), the effect of baseline self‐compassion on appearance concerns two and 6 months later was non‐significant. The results support and extend previous cross‐sectional research that has suggested a relationship between appearance concerns and psychological flexibility (or mindfulness, an element of psychological flexibility) in individuals following burn injuries (Shepherd et al., 2019) and other visible differences (Montgomery et al., 2016; Zucchelli, White, & Williamson, 2020). In line with the stress‐diathesis model of experiential avoidance (Biglan et al., 2008), individuals who typically have increased psychological flexibility may be more resilient in the event of appearance changes after burns. However, the findings of the current study do not fully support previous research proposing a role of self‐compassion in appearance concerns (Przezdziecki et al., 2013; Sherman et al., 2017; Todorov et al., 2019; Zhu et al., 2023).

Second, key factors that predict appearance concerns in the first 6 months post‐injury were identified. Male participants had lower appearance concerns compared to female participants at all time points, supporting previous research showing that females are more vulnerable to appearance concerns after burns (Lawrence et al., 2006; Shepherd et al., 2019; Thombs et al., 2008). Gender, perceived noticeability, psychological flexibility and self‐compassion predicted appearance concerns during hospital admission. The predictive value of perceived noticeability during hospital admission may reflect uncertainty about future appearance. Gender and psychological flexibility predicted appearance concerns two and six months later. However, when baseline appearance concerns were entered into the models, the effect of psychological flexibility diminished. Self‐compassion did not predict appearance concerns over time. These findings provide partial support for the hypothesis that increased levels of psychological flexibility and self‐compassion during hospital admission would predict lower levels of appearance concerns two and six months later.

Third, appearance concerns during hospital admission were the sole predictor of appearance concerns 2 months after hospital admission, and predicted appearance concerns 6 months later (in addition to gender). As stated above, when baseline appearance concerns were included in linear multiple regression analyses, the predictive value of psychological flexibility on subsequent appearance concerns diminished. This highlights the need for early psychological interventions to prevent or reduce appearance concerns after burns. It also emphasizes the need to identify psychological (resilience) factors that are associated with lower appearance concerns during hospital admission after a burn injury so these can be targeted in early psychological interventions.

Fourth, PTSD symptoms were linked to appearance concerns. Decreased PTSD symptoms were associated with lower appearance concerns cross‐sectionally at T1, T2 and T3. The association between appearance concerns and PTSD symptoms is consistent with previous research (Macleod et al., 2016) but has not been demonstrated previously in a prospective cohort study. This may be due to the burn injury serving as a visible reminder of the accident, thereby triggering PTSD symptoms (e.g. intrusive memories), or the development of negative appraisals associated with appearance changes (e.g. ‘My scars are ugly’), which maintains PTSD symptoms (Macleod et al., 2016).

Theoretically, the current findings enhance knowledge about the psychological variables associated with, and predictive of subsequent, appearance concerns following burn injuries. This evidence indicates that psychological flexibility is a protective factor against appearance concerns after burns. Clinically, this suggests that screening for psychological flexibility and appearance concerns during hospital admission may be useful to identify individuals at risk of appearance concerns over time. This may be particularly important for women given the current finding that gender predicts appearance concerns. The results also provide theoretical justification for the use of acceptance and commitment therapy (ACT; Hayes et al., 1999), which enhances psychological flexibility as an early psychological intervention to prevent and treat appearance concerns after burn injuries and possibly other visible difference populations. There is emerging evidence that ACT is acceptable and effective for individuals following burn injuries and other visible differences (Powell et al., 2023; Shepherd et al., 2020; Zucchelli et al., 2021; Zucchelli, Donnelly, et al., 2020). Our findings also imply that PTSD symptoms should be considered when assessing and treating appearance concerns following burn injuries.

### Strengths and limitations

The current findings should be considered in the context of several limitations. Although this is the first prospective cohort study of this nature to be conducted in the UK, the findings may lack generalizability to burn injury populations elsewhere or to those after the first 6 months post‐burn. To what extent the sample is representative of the burn injury population may be limited due to the inclusion/exclusion criteria and the convenience sampling employed, especially as the number of patients approached and declined participation was not recorded. In addition to controlling for PTSD symptoms, future studies would also benefit from controlling for more general measures of distress, such as anxiety and depression, which have been found to be elevated following burn injuries (Attoe & Pounds‐Cornish, 2015; Van Loey & Van Son, 2003). Furthermore, broader concepts related to compassion may be worthy of exploration in future research, such as compassion towards or from others and fear of compassion (Gilbert, 2014; Gilbert et al., 2011). Finally, the AAQ‐II, which was used to measure psychological flexibility, has recently been critiqued for lacking discriminant validity with measures of distress (Cherry et al., 2021). However, the AAQ‐II remains the most widely used measure of psychological flexibility, and research suggests that other measures are also not without limitations (Ong et al., 2020). Ong et al. (2020) also recommend the use of context‐specific measures of psychological flexibility where available. While specific measures of psychological flexibility in relation to body image are available, these are limited to weight/disordered eating and body dysmorphic disorder, which would not be appropriate to the context of individuals with visible burn injuries.

## CONCLUSIONS

Psychological flexibility is associated with appearance concerns in individuals after burn injuries, and therefore has a protective role against appearance concerns. Screening for psychological flexibility in addition to appearance concerns during hospital admission, while considering gender, may identify individuals at risk of appearance concerns over time. Further research should explore the effectiveness of ACT as an early psychological intervention for appearance concerns after burn injuries.

## AUTHOR CONTRIBUTIONS

**Laura Shepherd:** Conceptualisation; methodology; formal analysis; investigation; project administration; writing—original draft; writing—review and editing; visualisation; project administration; funding acquisition. **Fuschia M. Sirois:** Conceptualisation; methodology; formal analysis; writing—review and editing; visualisation; supervision. **Diana Harcourt:** Conceptualisation; methodology; formal analysis; writing—review and editing; visualisation; supervision. **Paul Norman:** Writing—review and editing; visualisation; supervision. **David Aaron:** Project administration; writing—original draft; writing—review and editing. **Kate Adkins:** Project administration; writing—original draft; writing—review and editing. **Anna Cartwright:** Project administration; writing—original draft; writing—review and editing. **Emma Hodgkinson:** Project administration; writing—original draft; writing—review and editing. **Nicola Murphy:** Project administration; writing—original draft; writing—review and editing. **Andrew R. Thompson:** Conceptualisation; methodology; formal analysis; writing—review and editing; visualisation; supervision. All authors have approved the final article to be submitted.

## CONFLICT OF INTEREST STATEMENT

Two authors, Andrew R. Thompson and Fuschia Sirois, are Co‐Editors of the journal.

## Supporting information


Appendix S1.

## Data Availability

The data that support the findings of this study are openly available in the Open Science Framework at https://doi.org/10.17605/OSF.IO/T8SJ4. Supplementary information and analyses are also available here.
