# Supplementary material for: A multi‐centre prospective cohort study investigating the roles of psychological flexibility and self‐compassion in appearance concerns after burn injuries
Source: Br J Health Psychol. 2024 Oct 2;30(1):e12754. doi: 10.1111/bjhp.12754 (PMC11586807; doi:10.1111/bjhp.12754)
Supplement: Supplementary file 1 — Appendix S1. [file BJHP-30-0-s001.docx]

Supplementary information

1. Additional variables not presented in the journal article

In addition to those reported in the journal article, the study collected data on two additional variables:

- Impairment in work and social functioning: The *Work and Social Adjustment Scale* (WSAS; Mundt et al., 2002), a five-item self-report questionnaire that measures impairment in work and social functioning, was completed at two time points (two months and six months after the hospital admission time point). Participants were prompted to answer this questionnaire based on the impact of any appearance concerns they were experiencing and to what extent they were interfering with their ability to: work; engage in home management tasks; participate in social leisure activities; complete private leisure activities; and form and maintain close relationships with other people. Responses to items fall on a scale ranging from 0 (‘Not at all’) to 8 (‘Very severely’). The total score of this measure has a possible range of values between 0 and 40. Higher scores indicate increased impairment in work and social functioning due to appearance concerns.
- Covid-19 distress: Distress related to the Covid-19 pandemic was also measured. Participants were prompted to state how distressed they were feeling about the Covid-19 pandemic on a scale of zero to 10 (where zero represented ‘Not at all’ and 10 represented ‘Extremely’). This was measured at all three time points (hospital admission, two months later and six months later).

*a) Means, SDs and ranges of scores on all data collected*

Table 1 presents means, SD and ranges of scores on all data collected.

**Table 1**

*Number of participants (N), means, standard deviation (SD) and ranges of data collected at each timepoint*

| Variable | *N* | *M* | *SD* | Range |
| --- | --- | --- | --- | --- |
| Appearance concerns | *T1:* 172  *T2:* 125  *T3:* 122 | *T1:* 2.65  *T2:* 2.47  *T3:* 2.45 | *T1:* 0*.*89  *T2:* 0.99  *T3:* 1.01 | *T1:* 0*.*1 – 4.0  *T2:* 0.2 – 4.0  *T3:* 0.1 – 4.0 |
| Impairment in work and social functioning | *T2:* 124  *T3:* 120 | *T2:* 8.16  *T3:* 7.03 | *T2:* 9.58  *T3:* 9.60 | *T2:* 0 - 40  *T3:* 0 - 38 |
| Psychological flexibility | *T1:* 175  *T2:* 125  *T3:* 121 | *T1:* 19.46  *T2:* 20.73  *T3:* 19.60 | *T1:* 11.15  *T2:* 11.62  *T3:* 10.46 | *T1:* 7 - 49  *T2:* 7 - 45  *T3:* 7 - 43 |
| Self-compassion | *T1:* 174  *T2:* 125  *T3:* 122 | *T1:* 3.35  *T2:* 3.04  *T3:* 3.07 | *T1:* 0.86  T2: 0.85  T3: 0.86 | *T1:* 1.33 – 5  *T2:* 1.3 – 5  *T3:* 1.1 – 5 |
| PTSD symptoms | *T1:* 174  *T2:* 125  *T3:* 121 | *T1:* 26.57  *T2:* 30.05  *T3:* 26.10 | *T1:* 21.04  *T2:* 20.72  *T3:* 19.57 | *T1:* 0 - 82  *T2:* 0 - 76  *T3:* 0 - 77 |
| Perceived noticeability scores | *T1:* 172  *T2:* 123  *T3:* 122 | *T1:* 2.40  *T2:* 2.49  *T3:* 2.11 | *T1:* 3.06  *T2:* 3.14  *T3:* 2.89 | *T1:* 0 - 10  *T2:* 0 - 10  *T3:* 0 - 10 |
| Covid-19 distress scores | *T1:* 175  *T2:* 124  *T3:* 121 | *T1:* 3.36  *T2:* 2.55  *T3:* 2.42 | *T1:* 3.00  *T2:* 2.72  *T3:* 2.72 | *T1:* 0-10  *T2:* 0-10  *T3:* 0-10 |

*b) Associations between demographic and clinical factors and the main study variables*

*Gender:* Independent t-tests revealed that female participants had increased appearance concerns compared to male participants at T1 (*t*(102.38) = 3.35, *p* = .001), T2 (*t*(123) = 3.10, *p* = .002) and T3 (*t*(120) = 3.55, *p* < .001). No differences were reported between men and women in impairment in work and social functioning due to appearance concerns at T2 (*t*(122) = -0.94, *p* = .35) or T3 (*t*(118) = -1.25, *p* = .21). An independent t-test revealed that at T1 there was no statistically significant difference in psychological flexibility between men and women at T1 (*t*(173) = -1.89, *p* = .06). Women had lower psychological flexibility at T2 (*t*(123) = -2.22, *p* = .028) and at T3 (*t*(119) = -2.38, *p* = .02). There were no statistically significant differences in self-compassion between men and women at T1 (*t*(172) = 1.15, *p* = .25), T2 (*t*(72.09) = 1.80, *p* = .08) or T3 (*t*(120) = 1.94, *p* = .06). Women had higher PTSD symptoms compared to men at T2 (*t*(123) = -2.74, *p* = .01) and T3 (*t*(119) = -1.82, *p* = .07) but not T1 (*t*(94.20) = -1.61, *p* = .11). No differences were found between men and women in perceived noticeability of the burns to other people at T1 (*t*(170) = -0.62, *p* = .54), T2 (*t*(121) = -.27, *p* = .79) or T3 (*t*(120) = -1.06, *p* = .29).

*Age*: Pearson’s correlation analyses revealed that at T1, there was no statistically significant relationship between age and appearance concerns (*r*(170) = .15, *p* = .06). There was a relationship between increased appearance concerns and younger age at T2 (*r*(123) = .20, *p* = .03) and T3 (*r*(120) = .20, *p* = .03). There was a relationship between younger age and increased impairment in work and social functioning due to appearance concerns at T2 (*r*(122) = -.19, *p* = .04) and T3 (*r*(118) = -.21, *p* = .03). There was an association between younger age and lower psychological flexibility at T1 (*r*(173) = -.18, *p* = .02), T2 *r*(123) = -.32, *p* < .001) and T3 (*r*(119) = -.27, *p* = .003). There was also a relationship between younger age and lower self-compassion at T1 (*r*(172) = .25, *p* < .001), T2 (*r*(123) = .23, *p* = .01) and T3 (*r*(119) = .27, *p* = .003). A significant relationship was also found between younger age and increased perceived noticeability of the burns to other people at T1 (*r*(170) = -.16, *p* = .03) but not at T2 (*r*(121) = -.13, *p* = .17) or T3 (*r*(120) = -.09, *p* = .35). There was an association between younger age and increased PTSD symptoms at T1 (*r*(172) = -.29, *p* < .001), T2 (*r*(123) = -.33, *p* < .001) and T3 (*r*(119) = -.31, *p* < .001).

*Ethnicity*: Independent t-tests reported no differences between participants from White compared to non-White ethnic backgrounds in relation to appearance concerns at T1 (*t*(167) = .80, *p* = .94), T2 (*t*(121) = -.46, *p* = .65) or T3 (*t*(13.78) = -.35, *p* = .73). No difference was reported between participants from White compared to non-White ethnic backgrounds in relation to impairment in work and social functioning due to appearance concerns at T2 (*t*(120) = -.66, *p* = .51) or T3 (*t*(116) = -.15, *p* = .88). Independent t-tests revealed no differences between participants from White compared to non-White ethnic backgrounds in relation to psychological flexibility at T1 (*t*(170) = -.28, *p* = .78), T2 (*t*(16.72) = .39, *p* = .71) or T3 (*t*(117) = .26, *p* = .80), or self-compassion at T1 (*t*(27.04) = -.46, *p* = .65), T2 (*t*(121) = -.37, *p* = .71) or T3 (*t*(118) = .11, *p* = .92). Participants from White ethnic backgrounds had lower PTSD symptoms at T2 (*t*(121) = -3.30, *p* = .01) compared to those from non-White backgrounds. No difference was reported between participants from White compared to non-White ethnic backgrounds in relation to PTSD symptoms at T1 (*t*(169) = -1.12, *p* = .27) or T3 (*t*(117) = -1.56, *p* = .12). No difference was reported between participants from White compared to non-White ethnic backgrounds in relation to perceived noticeability of the burns to other people at T1 (*t*(167) = .42, *p* = .68), T2 (*t*(119) = .01, *p* = 1.00) or T3 (*t*(118) = -1.12, *p* = .26).

*Socioeconomic status*: Pearson’s correlation analyses revealed a relationship between lower socioeconomic status and increased appearance concerns at T1 (*r*(169) = .20, *p* = .01), T2 (*r*(123) = .37, *p* < .001) and T3 (*r*(120) = .33, *p* < .001). There was an association between lower socioeconomic status and increased impairment in work and social functioning due to appearance concerns at T2 (*r*(122) = -.21, *p* = .02) but not T3 (*r*(118) = -.16, *p* = .09). Pearson’s correlational analyses found that lower socioeconomic status and lower psychological flexibility were related at T1 (*r*(172) = -.36, *p* < .001), T2 (*r*(123) = -.33, *p* < .001) and T3 (*r*(119) = -.25, *p* = .01). There was a relationship between lower socioeconomic status and lower self-compassion at T1 (*r*(171) = .33, *p* < .001), T2 (*r*(123) = .30, *p* < .001) and T3 (*r*(120) = .20, *p* = .03). There was no significant relationship between socioeconomic status and perceived noticeability of the burns to other people at T1 (*r*(169) = .09, *p* = .27), T2 (*r*(121) = -.06, *p* = .52) or T3 (*r*(120) = .02, *p* = .80). There was an association between lower socioeconomic status and increased PTSD symptoms at T1 (*r*(171) = -.15, *p* = .05) but not at T2 (*r*(123) = -.17, *p* = .06) or T3 (*r*(109) = -.15, *p* = .11).

*%TBSA*: Pearson’s correlation analyses revealed no significant relationship between appearance concerns and %TBSA at T1 (*r*(170) = -.070, *p* = .36), T2 (*r*(123) = -.07, *p* = .42). or T3 (*r*(120) = -.17, *p* = .065). There was no significant relationship between impairment in work and social functioning due to appearance concerns and %TBSA at T2 (*r*(122) = .13, *p* = .15) or T3 (*r*(118) = .05, *p* = .56). There was no relationship between %TBSA and perceived noticeability of the burns to other people at T1 (*r*(172) = .07, *p* = .36), T2 (*r*(123) = .08, *p* = .38) or T3 (*r*(122) = .03, *p* = .71). There was an association between %TBSA and PTSD symptoms at T1 (*r*(174) = .16, *p* = .04) but not at T2 (*r*(125) = .10, *p* = .26) or T3 (*r*(121) = .14, *p* = .13).

*Burn depth:* A between-participants ANOVA revealed a significant effect of burn depth on appearance concerns at T1 (*F*(3,168) = 4.74, *p* = .003), T2 (*F*(3,121) = 4.96, *p* = .003) and T3 (*F*(3,118) = 2.83, *p* = .04). Pairwise comparisons using Bonferroni adjustments revealed that at T1, appearance concerns were lower for participants with deep dermal burns compared to full thickness burns (mean difference = .89, *p* = .01) and deep dermal compared to mixed depth burns (mean difference = .92, *p* = .01). At T2, appearance concerns were lower for participants with superficial partial thickness burns compared to those with mixed depth (mean difference = .58, *p* = .03) and full thickness burns (mean difference = .72, *p* = .01). At T3, appearance concerns did not significantly differ according to depth of burns. A between-groups ANOVA revealed no significant main effect of burn depth on impairment in work and social functioning due to appearance concerns at T2 (*F*(3,120) = 1.59, *p* = .20) or T3 (*F*(3,116) = 2.31, *p* = .08). A between-groups ANOVA revealed a significant main effect of burn depth on perceived noticeability at T2 (*F*(3,119) = 3.15, *p* = .03) but not T1 (*F*(3,168) = 2.12, *p* = .10) or T3 (*F*(3,118) = 1.19, *p* = .32). At T2, pairwise comparisons using Bonferroni adjustments revealed that perceived noticeability was higher for participants with full thickness burns compared to deep dermal burns (mean difference = 3.39, *p* = .04). A between-groups ANOVA revealed no significant main effect of burn depth on PTSD symptoms at T1 (F(3,170) = 1.52, *p* = .21) or T2 (*F*(3,121) = 1.14, *p* = .34). However, there was a significant main effect of burn depth on PTSD symptoms at T3 (*F*(3,117) = 3.59, *p* = .02). At T3, pairwise comparisons using Bonferroni adjustments revealed that PTSD symptoms were higher for participants with full-thickness burns compared to superficial burns (mean difference = 13.20, *p* = .04), and higher for participants with mixed depth burns compared to superficial burns (mean difference = 11.09, *p* = .04).

*Location of the burn on the body*: Independent t-tests revealed that participants with burns to their head/face/neck compared to other areas of the body did not differ on appearance concerns at T1 (*t*(37.99) = -1.94, *p* = .06), T2 (*t*(123) = .55, *p* = .58) or T3 (*t*(120) = -.10, *p* = .92). No difference was reported between participants with burns to their head/face/neck compared to other body areas in relation to impairment in work and social functioning due to appearance concerns at T2 (*t*(122) = .02, *p* = .99) or T3 (*t*(118) = .01, *p* = .99). Participants with burns to their head/face/neck had higher levels of perceived noticeability at T1 (*t*(32.94) = 5.86, *p* < .001) and T2 (*t*(121) = 1.99, *p* = .05) but not T3 (*t*(120) = 1.47, *p* = .15) compared to participants with burns to other areas of their body. Independent t-tests revealed no difference between participants with burns to their hands compared to other areas of the body in relation to appearance concerns at T1 (*t*(170) = -.001, *p* = 1.00), T2 (*t*(123) = -.01, *p* = .99) or T3 (*t*(120) = -.02, *p* = .99). There was no difference in impairment in work and social functioning due to appearance concerns at T2 (*t*(122) = .81, *p* = .42) or T3 (*t*(42.15) = 1.56, *p* = .13) in those with burns to their hands compared to those with burns to other areas of the body. However, participants with burns to their hands had increased levels of perceived noticeability at T1 (*t*(170) = 2.26, *p* = .03) and T3 (*t*(42.44) = 2.52, *p* = .02) but not T2 (*t*(50.33) = 1.68, *p* = 1.00), compared to participants with burns to other areas of the body. Independent t-test revealed that participants with burns to their head/face/neck compared to other areas of the body did not differ on PTSD symptoms at T1 (*t*(172) = -.43, *p* = .67), T2 (*t*(123) = -.16, *p* = .87) or T3 (*t*(119) = .31, *p* = .76).

Between subjects t-tests comparing participants in the upper versus lower quartiles of appearance concerns as suggested in the protocol were considered superfluous given the correlation analyses. Moderation analyses were not considered appropriate given the sample size.

*c) Power analysis*

Two additional a priori power analyses were conducted, one for a correlational analysis and one for a multiple linear regression analysis. The power analysis for a correlational analysis was repeated to ensure consistency in the values used between the two power analyses. A power analysis using G*Power indicated that data from 29 participants would provide 80% power to detect a correlation of .50 between psychological flexibility and appearance anxiety at any of the time-points, based on a two-sided test with alpha = .05. Taking an estimate of retaining 67% of those recruited at follow-up, this suggested recruiting at least 43 participants.

*d) Analyses related to impairment in work and social functioning measure (Work and Social Adjustment Scale; WSAS; Mundt et al., 2002)*

A repeated measures t-test was conducted to compare the scores on the Work and Social Adjustment Scale (WSAS; Mundt et al., 2002) across the two data collection time points (two months and six months after hospital admission). This revealed that impairment in work and social functioning due to appearance concerns was higher at T2 compared to T3 (t(107) = 2.76, *p* = .01).

Pearson’s correlations were conducted to explore the relationships between scores on the primary and secondary dependent variables (appearance concerns and impact on work and social functioning), scores on the two independent variables (psychological flexibility and self-compassion) and pre-determined covariates (age, gender, ethnicity, % TBSA burnt, PTSD symptoms, perceived noticeability) at all three data collection time points (hospital admission, two months later and six months later). These analyses are presented in Table 2.

**Table 2**

*Relationships between appearance concerns and impairment in work and social functioning and psychological flexibility, self-compassion and the covariates*

|  | T1 Appearance concerns | T2 Appearance concerns | T3 Appearance concerns | T2 Impairment in work and social functioning | T3 Impairment in work and social functioning |
| --- | --- | --- | --- | --- | --- |

Gender -.26*** -.27*** -.31*** .09 .11

Age .15 .20* .20* -.19* -.21*

Ethnicity -.01 .04 .02 .06 .01

%TBSA -.07 -.07 -.17 .13 .05

T1 AAQ-II -.54*** -.51*** -.57*** .37*** .35***

T1 SCS-SF .50*** .45*** .51*** -.27** -.32***

T1 IES-R -.47*** -.41*** -.47*** .42*** .47***

T1 Perceived -.37*** -.20* -.21* .14 .24**

noticeability

T2 AAQ-II -.52*** -.73*** -.71*** .66*** .60***

T2 SCS-SF .53*** .68*** .69*** -.44*** -.41***

T2 IES-R -.39*** -.59*** -.55*** .62*** .61***

T2 Perceived -.16 -.32*** -.24** .43*** .44***

noticeability

T3 AAQ-II -.54*** -.72*** -.79*** .61*** .64***

T3 SCS-SF .52*** .66*** .70*** -.39*** -.41***

T3 IES-R -.41*** -.63*** -.61*** .67*** .70***

T3 Perceived -.18 -.38*** -.35*** .46*** .57***

noticeability

*_____________________________________________________________________*

*Note.* %TBSA: Percentage total body surface area burnt

AAQ-II: Acceptance and Action Questionnaire II (Bond et al., 2011) total score (higher scores indicate more difficulties with (i.e., lower) psychological flexibility

SCS-SF: Self-Compassion Scale-Short Form (Raes et al., 2011) mean total score (higher scores indicate increased self-compassion)

IES-R: Impact on Event Scale-Revised (Weiss & Marmar, 2007) total score (higher scores indicate increased PTSD symptoms)

* *p* < .05. ** *p* < .01. *** *p* < .001.

Partial correlation analyses were conducted to analyse the relationships between scores on the Appearance Subscale of the Body Esteem Scale for Adolescents and Adults (BESAA-A; Mendelson, Mendelson, & White, 2001), Work and Social Adjustment Scale (WSAS; Mundt et al., 2002), Acceptance and Action Questionnaire (AAQ-II; Bond et al., 2011) and Self-Compassion Scale – Short Form (SCS-SF; Raes et al., 2011) at each time point (hospital admission, two months later, six months later), controlling for pre-determined covariates measured at the same time point as the independent variables where applicable (i.e., PTSD symptoms and perceived noticeability). These are presented in Table 3.

**Table 3**

*Relationships between appearance concerns and impairment in work and social functioning and psychological flexibility and self-compassion, whilst controlling for covariates*

|  | T1 Appearance concerns | T2 Appearance concerns | T3 Appearance concerns | T2 Impairment in work and social functioning | T3 Impairment in work and social functioning |
| --- | --- | --- | --- | --- | --- |

T1 AAQ-II -.41*** -.35*** -.41*** .14 .11

T1 SCS-SF .40*** .26*** .36*** -.06 -.10

T2 AAQ-II -.55*** -.51*** .40*** .29**

T2 SCS-SF .55*** .58*** -.24** -.22*

T3 AAQ-II -.62*** .35***

T3 SCS-SF .62*** -.23*

*_____________________________________________________________________*

*Note.* AAQ-II: Acceptance and Action Questionnaire II (Bond et al., 2011) total score (higher scores indicate more difficulties with (i.e., lower) psychological flexibility

SCS-SF: Self-Compassion Scale-Short Form (Raes et al., 2011) mean total score (higher scores indicate increased self-compassion)

* *p* < .05. ** *p* < .01. *** *p* < .001.

Multiple linear regression analyses were conducted to explore how much of the variance in impairment in work and social functioning due to appearance concerns at T2 and T3 was explained by the covariates (block 1), T1 psychological flexibility and self-compassion (block 2) and T1 appearance concerns (block 3). These analyses are reported in Tables 4 and 5, respectively.

**Table 4**

*Hierarchical Regression Analysis Predicting Impairment in Work and Social Functioning at T2*

| Block 1 | | | | | |
| --- | --- | --- | --- | --- | --- |
|  | | | 95% CI | |  |
| Variable | B | SE | LL | UL | *p* |
| Gender | .082 | 1.794 | -3.473 | 3.638 | .964 |
| Perceived noticeability | .156 | .286 | -.411 | .723 | .587 |
| PTSD symptoms | .176 | .045 | .087 | .265 | <.001 |
| Age | -.056 | .056 | -.168 | .056 | .323 |
| Ethnicity | 1.589 | 3.143 | -4.640 | 7.818 | .614 |
| % TBSA | .060 | .103 | -1.44 | .264 | .562 |
| Block 2 | | | | | |
|  | | | 95% CI | |  |
| Variable | B | SE | LL | UL | *p* |
| Gender | -.237 | 1.792 | -3.790 | 3.316 | .895 |
| Perceived noticeability | .159 | .289 | -.414 | .732 | .584 |
| PTSD symptoms | .138 | .055 | .030 | .247 | .013 |
| Age | .-.057 | .057 | -.170 | .055 | .316 |
| Ethnicity | 1.687 | 3.187 | -4.630 | 8.003 | .598 |
| % TBSA | .063 | .103 | -.140 | .266 | .540 |
| Psychological flexibility | .254 | .147 | -.037 | .545 | .086 |
| Self-compassion | 1.788 | 1.862 | -1.903 | 5.479 | .339 |
| Block 3 | | | | | |
|  | | | 95% CI | |  |
| Variable | B | SE | LL | UL | *p* |
| Gender | -1.033 | 1.807 | -4.614 | 2.549 | .569 |
| Perceived noticeability | -.105 | .312 | -.723 | .513 | .737 |
| PTSD symptoms | .125 | .054 | .017 | .233 | .024 |
| Age | -.060 | .056 | -.171 | .051 | .288 |
| Ethnicity | 1.841 | 3.140 | -4.384 | 8.065 | .559 |
| % TBSA | .073 | .101 | -.127 | .274 | .470 |
| Psychological flexibility | .148 | .154 | -.157 | .452 | .338 |
| Self-compassion | 1.649 | 1.835 | -1.990 | 5.288 | .371 |
| T1 Appearance concerns | -2.549 | 1.229 | -4.985 | -.114 | .040 |

When exploring the impact of the variables on T2 impairment in work and social functioning, the analysis revealed that the covariates explained 18.9% of the variance in T2 impairment in work and social functioning (*R*^2^ = .19). The model was statistically significant (*F*(6,110) = 4.27, *p* < .001). Only PTSD symptoms were a statistically significant predictor. Adding in T1 psychological flexibility and self-compassion explained a further 2.4% of the variance (Δ*R*^2^ = .02, Δ*F* = 1.66, *p* = .20) and this model was statistically significant (*F*(8,108) = 3.65, *p* < .001). In this model, PTSD symptoms continued to be the only statistically significant predictor. Adding in T1 appearance concerns explained a further 3% of the variance (Δ*R*^2^ = .03, Δ*F* = 4.31, *p* = .04) and this model was statistically significant (*F*(9,107) = 3.83, *p* < .001). In this model, T1 PTSD symptoms continued to be a significant predictor along with T1 appearance concerns.

**Table 5**

*Hierarchical Regression Analysis Predicting Impairment in Work and Social Functioning at T3*

| Block 1 | | | | | |
| --- | --- | --- | --- | --- | --- |
|  | | | 95% CI | |  |
| Variable | B | SE | LL | UL | *p* |
| Gender | .272 | 1.782 | -3.261 | 3.805 | .879 |
| Perceived noticeability | .386 | .280 | -.169 | .940 | .171 |
| PTSD symptoms | .210 | .044 | .123 | .296 | <.001 |
| Age | -.041 | .056 | -.152 | .071 | .470 |
| Ethnicity | -1.322 | 3.147 | -7.562 | 4.917 | .675 |
| % TBSA | -.041 | .102 | -.242 | .161 | .689 |
| Block 2 | | | | | |
|  | | | 95% CI | |  |
| Variable | B | SE | LL | UL | *p* |
| Gender | .185 | 1.813 | -3.410 | 3.780 | .919 |
| Perceived noticeability | .415 | .284 | -.148 | .977 | .147 |
| PTSD symptoms | .178 | .052 | .074 | .281 | <.001 |
| Age | -.034 | .057 | -.148 | .080 | .554 |
| Ethnicity | -1.033 | 3.170 | -7.318 | 5.252 | .745 |
| % TBSA | -.049 | .103 | -.252 | .154 | .633 |
| Psychological flexibility | .075 | .145 | -.212 | .363 | .605 |
| Self-compassion | -.436 | 1.841 | -4.087 | 3.216 | .813 |
| Block 3 | | | | | |
|  | | | 95% CI | |  |
| Variable | B | SE | LL | UL | *p* |
| Gender | -.013 | 1.848 | -3.676 | 3.651 | .995 |
| Perceived noticeability | .328 | .318 | -.303 | .959 | .305 |
| PTSD symptoms | .175 | .053 | .070 | .279 | <.001 |
| Age | -.034 | .057 | -.148 | .080 | .552 |
| Ethnicity | -.987 | 3.180 | -7.294 | 5.319 | .757 |
| % TBSA | -.046 | .103 | -.250 | .158 | .655 |
| Psychological flexibility | .049 | .152 | -.251 | .350 | .745 |
| Self-compassion | -.354 | 1.852 | -4.026 | 3.318 | .849 |
| T1 Appearance concerns | -.743 | 1.223 | -3.168 | 1.681 | .545 |

In predicting T3 impairment in work and social functioning, the covariates explained 24.7% of the variance in T3 impairment in work and social functioning due to appearance concerns (*R*^2^ = .25). The model was statistically significant (*F*(6,107) = 5.84, *p* < .001). Only PTSD symptoms were a statistically significant predictor. Adding in T1 psychological flexibility and self-compassion explained a further 0.9% of the variance (Δ*R*^2^ = .01, Δ*F* = 0.64, *p* = .53) and this model was statistically significant (*F*(8,105) = 4.51, *p* < .001). In this model, PTSD symptoms remained the only statistically significant predictor. Adding in T1 appearance concerns explained a further 0.3% of the variance (Δ*R*^2^ = .003, Δ*F* = 0.37, *p* = .55) and this model was statistically significant (*F*(9,104) = 4.02, *p* < .001). In this model, PTSD symptoms remained the only statistically significant predictor.

*e) Analyses related to Covid-19 distress measure*

A repeated measures ANOVA revealed that Covid-19 distress changed over time (*F*(2,216) = 12.64, *p* < .001), with distress being higher at T1 compared to both T2 (mean difference = .86, *p* = .002) and T3 (mean difference = 1.15, *p* < .001).

Pearson correlation analyses revealed that increased Covid-19 distress was significantly related to increased appearance concerns at T1 (*r* = -.27, *n* = 172, *p* < .001), T2 (*r* = -.28, *n* = 124, *p* = .002) and T3 (*r* = =.31, *n* = 121, *p* < .001), lower psychological flexibility at T1 (*r* = .280, *n* = 175, *p* < .001) and T3 (*r* = .22, *n* = 120, *p* = .02) but not T2 (*r* = .13, *n* = 149, *p* = .15), and lower self-compassion at T1 (*r* = -.20, *n* = 174, *p* = .01) and T2 (*r* = -.18, *n* = 124, *p* = .04) but not T3 (*r* = -.13, *n* = 121, *p* = .15).

Partial correlation analyses were conducted to explore the relationships between appearance concerns, psychological flexibility and self-compassion, when controlling for the a priori covariates in addition to Covid-19 distress. These are displayed in Table 6 and show that the relationships between increased appearance concerns and lower psychological flexibility and self-compassion cross-sectionally at all time points and prospectively across time were maintained when Covid-19 distress was controlled.

**Table 6**

*Relationships between appearance concerns and impairment in work and social functioning and psychological flexibility and self-compassion, whilst controlling for covariates and Covid-19 distress*

|  | T1 Appearance concerns | T2 Appearance concerns | T3 Appearance concerns |
| --- | --- | --- | --- |

T1 AAQ-II -.40*** -.36*** -.41***

T1 SCS-SF .39*** .26** .36***

T2 AAQ-II -.57*** -.54***

T2 SCS-SF .55*** .58***

T3 AAQ-II -.63***

T3 SCS-SF .62***

*____________________________________________________*

*Note.* AAQ-II: Acceptance and Action Questionnaire II (Bond et al., 2011) total score (higher scores indicate more difficulties with (i.e., lower) psychological flexibility.

SCS-SF: Self-Compassion Scale-Short Form (Raes et al., 2011) mean total score (higher scores indicate increased self-compassion)

* *p* < .05. ** *p* < .01. *** *p* < .001.

Multiple regression analyses to explore the predictors of appearance concerns at T1, T2 and T3 were repeated with the inclusion of Covid-19 distress. The covariates in addition to Covid-19 distress (block 1), T1 psychological flexibility and self-compassion (block 2) and T1 appearance concerns (block 3) were entered hierarchically. Tables 7 to 9 present the predictors for each of these analyses at each of the three time points.

**Table 7**

*Hierarchical Regression Analysis Predicting Appearance Concerns at T1*

| Block 1 | | | | | | | | | | |  |
| --- | --- | --- | --- | --- | --- | --- | --- | --- | --- | --- | --- |
|  | | | | | 95% CI | | | |  | |  |
| Variable | B | | SE | | LL | | UL | | *p* | |  |
| Gender | -.370 | | 1.25 | | -.616 | | -.124 | | .003 | |  |
| Perceived noticeability | -.081 | | .019 | | -.119 | | -.043 | | <.001 | |  |
| PTSD symptoms | -.014 | | .003 | | -.020 | | -.008 | | <.001 | |  |
| Age | .002 | | .004 | | -.006 | | .010 | | .672 | |  |
| Ethnicity | .203 | | .203 | | -.198 | | .604 | | .319 | |  |
| % TBSA | .002 | | .007 | | -.013 | | .016 | | .828 | |  |
| Covid-19 distress | -.046 | | .021 | | -.088 | | -.004 | | .031 | |  |
| Block 2 | | | | | | | | | | |  |
|  | | | | | 95% CI | | | |  | |  |
| Variable | B | | SE | | LL | | UL | | *p* | |  |
| Gender | -.332 | | .114 | | -.547 | | -.097 | | .005 | |  |
| Perceived noticeability | -.098 | | .018 | | -.134 | | -.063 | | <.001 | |  |
| PTSD symptoms | -.003 | | .003 | | -.010 | | .003 | | .311 | |  |
| Age | -.001 | | .004 | | -.008 | | .007 | | .872 | |  |
| Ethnicity | .052 | | .187 | | -.319 | | .422 | | .784 | |  |
| % TBSA | .003 | | .007 | | -.011 | | .016 | | .678 | |  |
| Covid-19 distress | -.028 | | .019 | | -.067 | | .010 | | .150 | |  |
| Psychological flexibility | | -.021 | | .008 | | -0.037 | | -.005 | | .012 | |
| Self-compassion | .224 | | .102 | | .023 | | .426 | | .030 | |  |

At T1, the analysis revealed that the covariates explained 35% of the variance (*R*^2^ = .35). The model was statistically significant (*F*(7,164) = 12.07, *p* < .001). Male gender, decreased perceived noticeability, PTSD symptoms and Covid-19 distress significantly predicted lower appearance concerns. Adding in T1 psychological flexibility and self-compassion explained a further 11.9% of the variance in T2 appearance concerns (Δ*R*^2^ = .12, Δ*F* = 17.41, *p* < .001). This model was also statistically significant (*R*^2^ = .47, *F*(9,164) = 15.22, *p* < .001). Male gender, decreased perceived noticeability and increased psychological flexibility and self-compassion significantly predicted lower appearance concerns.

**Table 8**

*Hierarchical Regression Analysis Predicting Appearance Concerns at T2*

| Block 1 | | | | | |
| --- | --- | --- | --- | --- | --- |
|  | | | 95% CI | |  |
| Variable | B | SE | LL | UL | *p* |
| Gender | -.444 | .178 | -.797 | -.091 | .014 |
| Perceived noticeability | -.023 | .029 | -.080 | .033 | .416 |
| PTSD symptoms | -.017 | .005 | -.026 | -.007 | <.001 |
| Age | .006 | .006 | -.006 | .018 | .330 |
| Ethnicity | .384 | .317 | -.243 | 1.011 | .228 |
| % TBSA | .000 | .010 | -.020 | .021 | .983 |
| Covid-19 distress | .006 | .031 | -.055 | .068 | .841 |
| Block 2 | | | | | |
|  | | | 95% CI | |  |
| Variable | B | SE | LL | UL | *p* |
| Gender | -.392 | .168 | -.725 | -.059 | .022 |
| Perceived noticeability | -.035 | .027 | -.090 | .019 | .199 |
| PTSD symptoms | -.006 | .005 | -.016 | .005 | .282 |
| Age | .004 | .006 | -.008 | .015 | .519 |
| Ethnicity | .241 | .304 | -.362 | .844 | .430 |
| % TBSA | .000 | .010 | -.019 | .020 | .962 |
| Covid-19 distress | .015 | .029 | -.043 | .073 | .610 |
| Psychological flexibility | -.038 | .014 | -.065 | -.010 | .007 |
| Self-compassion | -.029 | .176 | -.377 | .319 | .869 |
| Block 3 | | | | | |
|  | | | 95% CI | |  |
| Variable | B | SE | LL | UL | *p* |
| Gender | -.248 | .156 | -.558 | .061 | .114 |
| Perceived noticeability | .015 | .027 | -.039 | .069 | .584 |
| PTSD symptoms | -.004 | .005 | -.014 | .006 | .405 |
| Age | .002 | .005 | -.008 | .013 | .633 |
| Ethnicity | .177 | .277 | -.373 | .727 | .524 |
| % TBSA | -.001 | .009 | -.019 | .017 | .914 |
| Covid-19 distress | .042 | .027 | -.013 | .096 | .130 |
| Psychological flexibility | -.016 | .013 | -.042 | .011 | .248 |
| Self-compassion | .009 | .160 | -.309 | .327 | .956 |
| T1 Appearance concerns | .526 | .109 | .309 | .742 | <.001 |

At T2, the covariates explained 22.5% of the variance in T2 appearance concerns (*R*^2^ = .23). The model was statistically significant (*F*(7,117) = 4.574, *p* < .001). Male gender and decreased PTSD symptoms significantly predicted lower appearance concerns. Adding in T1 psychological flexibility and self-compassion explained a further 10.1% of the variance (Δ*R*^2^ = .101, Δ*F* = 8.131, *p* < .001) and this model was statistically significant (*F*(9,117) = 5.826, *p* < .001). In this model, male gender and increased psychological flexibility significantly predicted lower appearance concerns. Adding in T1 appearance concerns explained a further 12% of the variance (Δ*R*^2^ = .12, Δ*F* = 23.150, *p* < .001) and this model was statistically significant (*F*(10,117) = 8.634, *p* < .001). In this model, only lower T1 appearance concerns significantly predicted lower T2 appearance concerns.

**Table 9**

*Hierarchical Regression Analysis Predicting Appearance Concerns at T3*

| Block 1 | | | | | |
| --- | --- | --- | --- | --- | --- |
|  | | | 95% CI | |  |
| Variable | B | SE | LL | UL | *p* |
| Gender | -.554 | .179 | -.908 | -.199 | .003 |
| Perceived noticeability | -.017 | .028 | -.073 | .039 | .553 |
| PTSD symptoms | -.018 | .005 | -.028 | -.009 | <.001 |
| Age | .003 | .006 | -.009 | .014 | .652 |
| Ethnicity | .438 | .325 | -.207 | 1.083 | .181 |
| % TBSA | -.013 | .010 | -.033 | .007 | .206 |
| Covid-19 distress | -.008 | .031 | -.070 | .055 | .805 |
| Block 2 | | | | | |
|  | | | 95% CI | |  |
| Variable | B | SE | LL | UL | *p* |
| Gender | -.526 | .166 | -.856 | -.197 | .002 |
| Perceived noticeability | -.029 | .026 | -.081 | .023 | .272 |
| PTSD symptoms | -.006 | .005 | -.016 | .004 | .201 |
| Age | .000 | .006 | -.011 | .011 | .936 |
| Ethnicity | .310 | .299 | -.283 | .904 | .303 |
| % TBSA | -.010 | .009 | -.028 | .008 | .283 |
| Covid-19 distress | .001 | .029 | -.057 | .058 | .985 |
| Psychological flexibility | -.030 | .013 | -.057 | -.003 | .027 |
| Self-compassion | .162 | .171 | -.177 | .502 | .346 |
| Block 3 | | | | | |
|  | | | 95% CI | |  |
| Variable | B | SE | LL | UL | *p* |
| Gender | -.378 | .150 | -.675 | -.080 | .013 |
| Perceived noticeability | .033 | .026 | -.019 | .084 | .211 |
| PTSD symptoms | -.006 | .004 | -.014 | .003 | .213 |
| Age | -.002 | .005 | -.012 | .008 | .709 |
| Ethnicity | .200 | .267 | -.328 | .729 | .454 |
| % TBSA | -.010 | .008 | -.027 | .006 | .217 |
| Covid-19 distress | .033 | .026 | -.020 | .085 | .221 |
| Psychological flexibility | -.010 | .012 | -.035 | .014 | .414 |
| Self-compassion | .124 | .152 | -.178 | .426 | .418 |
| T1 Appearance concerns | .549 | .102 | .347 | .751 | <.001 |

At T3, the covariates explained 29.3% of the variance in T3 appearance concerns (*R*^2^ = .29). The model was statistically significant (*F*(7,114) = 6.333, *p* < .001). Male gender and decreased PTSD symptoms significantly predicted lower appearance concerns. Adding in T1 psychological flexibility and self-compassion explained a further 12.6% of the variance (Δ*R*^2^ = .13, Δ*F* =11.43, *p* < .001) and this model was statistically significant (*F*(9,114) = 8.428, *p* < .001). In this model, male gender and increased psychological flexibility significantly predicted lower appearance concerns. Adding in T1 appearance concerns explained a further 12.7% of the variance (Δ*R*^2^ = .13, Δ*F* =28.991, *p* < .001) and this model was statistically significant (*F*(10,114) = 12.506, *p* < .001). In this model, male gender and lower T1 appearance concerns significantly predicted lower T3 appearance concerns.

References

Bond, F. W., Hayes, S. C., Baer, R. A., Carpenter, K. M., Guenole, N., Orcutt, H. K., Waltz,

T., & Zettle, R. D. (2011). Preliminary psychometric properties of the Acceptance

and Action Questionnaire–II: A revised measure of psychological inflexibility and experiential avoidance. *Behavior Therapy*, *42*(4), 676–688. [https://doi:10.1016/j.beth.2011.03.007](about:blank)

Mendelson, B. K., Mendelson, M. J., & White, D. R. (2001). Body-esteem scale for adolescents and adults. *Journal of Personality Assessment, 76*(1), 90-106.

<https://doi.org/10.1207/S15327752JPA7601_6>

Mundt, J. C., Marks, I. M., Shear, M. K., & Greist, J. H. (2002). The Work and Social Adjustment Scale: a simple measure of impairment in functioning. *The British Journal of Psychiatry*, *180*, 461–464. <https://doi.org/10.1192/bjp.180.5.461>

Raes, F., Pommier, E., Neff, K. D., & Van Gucht, D. (2011). Construction and factorial

validation of a short form of the Self-Compassion Scale. *Clinical Psychology & Psychotherapy, 18*(3)*,* 250-255. <https://doi.org/10.1002/ccp.702>

Weiss, D. S., & Marmar, C. R. (1997). The Impact of Event Scale- Revised. In: Wilson JP,

Keane TM, editors. *Assessing psychological trauma and PTSD.* New York: Guilford; p. 399- 411.
